# Awareness of Post-COVID-19 Syndrome Among the General Population of the Kingdom of Saudi Arabia

**DOI:** 10.7759/cureus.64582

**Published:** 2024-07-15

**Authors:** Mushabab Alghamdi, Sami Mohammed A Alaklabi, Saud G Alshmrani, Yaser Mohammed D Alamri, Turki A Alamri, Turki M Alaklabi, Sultan Saber Z Alharethi, Salem Mohammed K Alalyani, Masoud I E Adam

**Affiliations:** 1 Department of Internal Medicine, University of Bisha, Bisha, SAU; 2 College of Medicine, University of Bisha, Bisha, SAU; 3 Department of Medicine: Medical Education, University of Bisha, Bisha, SAU

**Keywords:** covid-19, covid-19 vaccine, awareness, risk factors, post covid syndrome

## Abstract

Background

Post-COVID-19 syndrome (PC19S) is an emerging pathological entity characterized by the development or persistence of a spectrum of symptoms and signs 12 weeks after the original disease. Most COVID-19 patients show a variety of persistent symptoms after recovery that impact their quality of life and professional performance. The prevalence of PC19S is found to be high among many populations hence, the need for knowledge and understanding of its risk factors, symptoms, and the awareness of the population about them to improve the provided health and medical care.

Aim

This study aims to assess the level of awareness of post-COVID-19 syndrome among the general population of the Kingdom of Saudi Arabia (KSA). Most studies have focused on hospitalized patients and those with severe disease, but PC19S can exist in other categories of COVID-19 patients; hence, the need for total population coverage.

Methodology

A cross-sectional study was conducted during the period between June 2023 and August 2023 using a structured self-administered online questionnaire. The online questionnaire in addition to the demographic characteristics consists of two main parts, one is about the awareness of the Saudi population of symptoms of PC19S and the other is about awareness of its risk factors.

Results

The majority of the participants (1558; 72.4%) showed low awareness of PC19S symptoms while only about one quarter (595; 27.6%) showed satisfactory awareness. Also, the awareness of the participants toward risk factors was low, as 1738 (80.7%) of them showed low awareness. We categorized the results into three levels of awareness to simplify and facilitate interpretation. The findings showed that 1380 individuals (64.09%) had low awareness of PC19S, 536 individuals (24.89%) had moderate awareness, and only 237 individuals (11%) had high awareness. The study reported that the highest awareness toward symptoms was of smell disturbances (1206; 56.0%) and the least was of hair loss (506; 23.5%) while among the risk factors, the highest was found toward old age 1326 (61.6%) and the female sex was the lowest 194 (9.0%).

Conclusion

The study revealed that the majority of the participants demonstrated low awareness of symptoms and risk factors, which needs a continuous effort to raise the population's awareness of this health-threatening condition.

## Introduction

COVID-19 is primarily an acute, contagious, respiratory viral disease caused by severe acute respiratory syndrome coronavirus 2 (SARS‑CoV‑2) but can involve multiple organs [1,2]. The disease has immediate complications, mainly respiratory, but can also cause acute cardiac complications or thrombotic disease during the acute phase. Delayed complications have also been observed after recovery [3,4]. Some patients who recovered from a COVID-19 infection during the first wave developed persistent symptoms, which could be due to the virus itself or other factors [5]. The developing symptoms come in different severities [6].^ ^In 2020, post-COVID syndrome was first described during a survey of prolonged COVID-19 symptoms [7]. It is reported that 40%-70% of those who survived COVID-19 may develop post-COVID-19 syndrome (PC19S) [8]. Studies suggest a more conservative estimate, indicating that around 10% to 20% of people infected by SARS-CoV-2 may develop symptoms that can be diagnosed as long COVID [9].

The World Health Organization (WHO) has given the names post-COVID-19 condition and long COVID and refers to it as “the continuation or development of new symptoms 3 months after the initial SARS-CoV-2 infection, with these symptoms lasting for at least 2 months with no other explanation,” which “can affect anyone exposed to SARS-CoV-2, regardless of age or severity of original symptoms” [9]. The Centers for Disease Control (CDC) used the name "post-Covid conditions" for illnesses that persist more than four weeks after the primary disease [3].

During the pandemic, the prevalence of COVID-19 in KSA ranged from 1.78% to 24.45% in different regions of the country [10]. The Saudi Ministry of Health defined post-COVID-19 syndrome as a ‘‘spectrum of signs and symptoms that persist for or develop after 12 weeks following an acute COVID-19 infection and are not explained by an alternative diagnosis, which should be assessed and excluded’’ [11]. PC19S is rare in children and adolescents but commonly seen in adults and can develop in all categories of COVID patients, PC19S can affect those who have no symptoms, those with mild symptoms, and those with severe COVID-19 disease [12].

A multifactorial pathophysiological origin is believed to be the cause of post-COVID-19 syndrome [13]. Incriminated factors include microvascular ischemia, injury, immobility, and metabolic alterations that take place during the acute phase of the disease [14]. PC19S has a great impact on individual performance and work ability [15].

The prevalence of PC19S among Saudis varies in different areas due to many factors but according to the Saudi Ministry of Health, 96% of COVID-19 patients had not returned to their usual state of health when interviewed at 90 days (3 months) later [11]. According to Hyassat D et al., the prevalence of post-COVID-19 syndrome symptoms was 59.3% [16]. A similar finding was reported by Fernández-de-Las-Peñas et al. of COVID-19 survivors (60%) [17] while some studies reported higher prevalence rates, such as Huang et al. (76%) [18], Taboada et al. (67%) [19], and Carfi et al. (87%) [20]. Many studies have reported that PC19S is more common among females than males [18,21,22].

Post-COVID conditions were highly prevalent among adults in Saudi Arabia (74.60%) [23]. Females, young adults, and those with higher stress levels were more likely to experience post-COVID conditions compared to males and middle-aged adults, respectively, according to a recent study carried out by Ashgar R [23].

According to the Saudi National Guidelines in Post-COVID-19 Clinical Care by the Ministry of Health, Saudi Arabia (2022), the most common COVID-19 symptoms include fatigue, musculoskeletal symptoms, dyspnea and respiratory complications, cardiovascular abnormalities, neurological impairment, gastrointestinal and hepatic impairment, psychological impairments and post-traumatic stress disorders, metabolic impairment, post-viral olfactory dysfunction, post-viral taste dysfunction, post-viral menstrual irregularities, and long-term cognitive impairments [11]. PC19S is a group of symptoms affecting individual health and involving many risk factors. Due to limited research on this subject, this study aims to bridge the gap in knowledge and awareness, providing valuable information to educate the population about this syndrome and determine the level of awareness about PC19S, its symptoms, risk factors, associated risks, and factors influencing awareness among the general population of Saudi Arabia.

## Materials and methods

Our research is a community-based, cross-sectional study conducted in the Kingdom of Saudi Arabia (KSA). The target demographic comprised adults residing in KSA, inclusive of all males and females aged 18 years and older, who have lived in the country for a minimum of six months. Individuals who declined participation or did not meet the inclusion criteria were excluded.

Data collection was executed via an online, standardized, self-administered questionnaire, which was developed by the researchers and reviewed by two consultants (Appendices). To address potential language barriers, a back-translation process was implemented. This involved translating the English version of the questionnaire into Arabic by a professional translator and then translating it back into English to ensure the equivalence of meaning between the original and translated versions.

The questionnaire was divided into three sections. The first section gathered socio-demographic information, including age, gender, marital status, educational level, residency, nationality, occupation, income, and personal and family history of COVID-19. The second section consisted of nine close-ended questions designed to assess knowledge about the symptoms of PC19S. The third section comprised eight questions aimed at evaluating participants' knowledge regarding the risk factors of PC19S.

A non-probability convenience sampling technique was employed. Collaboration with 26 data collectors from various regions of KSA was sought, with each data collector tasked to recruit at least 100 participants. Ultimately, 16 data collectors successfully gathered the required samples. They utilized diverse distribution methods such as WhatsApp, Telegram, Facebook, and Twitter.

Data were collected through Google Forms (Google LLC, Mountain View, California, US) and subsequently exported to Microsoft Excel 2010 (Microsoft Corporation, Redmond, WA, US) for encoding and analysis using the Statistical Package for Social Sciences (SPSS) version 26. Basic variables were expressed as frequency tables, and data were presented in percentages, depicted through tables. The chi-square test was used for the analysis of categorical data, with results considered statistically significant at a p-value of less than 0.05. The awareness of PC19S was assessed by making a cut-point of 50% (5 or more out of 9 known symptoms considered as high awareness level while less than 5 considered as low level of awareness, and 4 or more out of 8 known risk factors are considered as high awareness level while less than 5 considered as low level of awareness). We adjusted the results and reorganized them into levels of awareness as low awareness level regarding PC19S (has a low level of awareness regarding both symptoms and risk factors), moderate awareness level regarding PC19S (has a high level of awareness regarding either symptoms or risk factors) and high awareness level regarding PC19S (has a high level of awareness regarding both symptoms and risk factors), which were to simplify and facilitate the results’ interpretation.

Ethical approval was obtained from the University of Bisha Institutional Review Board (IRB) before collecting data. Participants were informed about the study's objectives, and consent was obtained before they started the questionnaires. Participants were also informed of their right to withdraw from the study at any time. Confidentiality was maintained throughout the study, ensuring that the respondents' information was secured and kept private.

## Results

The study was conducted among the whole Saudi population excluding those below 18 years (Table 1) and involved 2153; out of them, 907 (42.13%) had been infected with COVID-19, 1035 (48.07%) were males, 1118 (51.93%) females, and most of them are Saudi 2061 (95.73%). Nearly half of the participants were single 1041 (48.4%) while 961 (44.6%) were married and the remaining were divorced (77; 3.6%), widows (46; 2.1%), and separated (28; 1.3%). Bachelor's degree/diploma holders constituted almost two-thirds of the participants (1353; 62.8%) while high school (577; 26.8%), intermediate school (48; 2.2%), elementary school (18; 0.8%), graduate degree holders (133; 6.2%), and those who had no formal education (24; 1.1%). More than one-third of the participants were from the northern region (777; 36.09%) followed by the southern region (466; 21.64%), western region (414; 19.23%), middle region (340; 15.79%), and the least from the eastern region (156; 7.25%). Unemployed participants were 918 (42.6%) while educational sector employees were 414 (19.2%), health sector workers were 286 (13.3%), private companies’ employees (251; 11.7%), military service personnel (165; 7.7% and 119; 5.5%). Regarding the income of the participants, those with the lowest income constitute more than half of them (1089; 50.6%) while the highest and those with a modest income together less than half of the participants (550; 25.5%) and (514; 23.9%), respectively.

**Table 1:** Characteristics of the participants This table represents the data in N, with its percentage (%), chi-square value, and p-value. *Significant (p ≤ 0.05); **Non-significant (p > 0.05); N: number

Parameter	Category	N (%)	Chi-square test (N 2153)	P-value
Age	18-36 years old	1429 (66.37%)	2.686	0.612**
	37-56 years old	637 (29.59%)		
	+56 years old	87 (4.04%)		
Gender	Male	1035 (48.07%)	26.150	0.001*
	Female	1118 (51.93%)		
Nationality	Saudi	2061 (95.73%)	1.781	0.410**
	Non-Saudi	92 (4.27%)		
Marital status	Single	1041 (48.4%)	15.912	0.044*
	Married	961 (44.6%)		
	Divorced	77 (3.6%)		
	Widower	46 (2.1%)		
	Separated	28 (2.3%)		
Educational level	No formal education	24 (1.1%)	14.437	0.154**
	Elementary school	18 (0.8%)		
	Intermediate school	48 (2.2%)		
	High school	577 (26.8%)		
	Bachelor’s degree/diploma	1353 (62.8%)		
	Postgraduate degree	133 (6.2%)		
Residence	Northern region	777 (36.09%)	55.695	0.001*
	Southern region	466 (21.64%)		
	West region	414 (19.23%)		
	Middle region	340 (15.79%)		
	East region	156 (7.25%)		
Occupation	Education sector	414 (19.2%)	35.952	0.001*
	Health sector	286 (13.3%)		
	Private companies	251 (11.7%)		
	Military service	165 (7.7%)		
	Unemployed	918 (42.6%)		
	Retired	119 (5.5%)		
Income per month	Less than 5000 SAR (1333 USD)	1089 (50.6%)	5.996	0.199**
	5000-10000 SAR (1333-2666 USD)	514 (23.9%)		
	More than 10000 SAR (2666 USD)	550 (25.5%)		
Personal history of COVID-19 infection	Yes	907 (42.13%)	4.443	0.108**
	No	1246 (57.87%)		
Family history of COVID-19 infection	Yes	1453 (67.49%)	19.759	0.001*
	No	700 (32.51%)		

We adjusted the results and reorganized them into three levels of awareness as shown in Table 2. This was to simplify and facilitate the results’ interpretation, which revealed 1380 (64.09%) with low awareness of PC19S, 536 (24.89%) with moderate awareness of PC19S, and only 237 (11%) with high awareness level.

**Table 2:** Sociodemographic parameters and levels of awareness This table represents the data in N: num with its percentage (%).

Parameter	Category	N (%)	Low awareness level regarding PC19S (Has a low level of awareness regarding both symptoms and risk factors)	Moderate awareness level regarding PC19S (Has a high level of awareness regarding either symptoms or risk factors)	High awareness level regarding PC19S (Has a high level of awareness regarding both symptoms and risk factors)
Age	18-36 Years old	1429 (66.37%)	917 (64.17%)	364 (25.47%)	148 (10.35%)
	37-56 Years old	637 (29.59%)	405 (63.57%)	152 (23.86%)	80 (12.55%)
	+56 Years old	87 (4.04%)	58 (66.66%)	20 (22.98%)	9 (10.34%)
Gender	Male	1035 (48.07%)	719 (69.46%)	226 (21.83%)	90 (8.69%)
	Female	1118 (51.93%)	661 (59.12%)	310 (27.72%)	147 (13.14%)
Nationality	Saudi	2061 (95.73%)	1323 (64.19%)	515 (24.98%)	223 (10.81%)
	Non-Saudi	92 (4.27%)	57 (61.95%)	21 (22.82%)	14 (15.21%)
Marital status	Single	1041 (48.4%)	643 (61.76%)	275 (26.41%)	123 (11.81%)
	Married	961 (44.6%)	631 (65.66%)	236 (24.55%)	94 (9.78%)
	Divorced	77 (3.6%)	50 (64.93%)	16 (20.77%)	11 (14.28%)
	Widower	46 (2.1%)	37 (80.43%)	6 (13.04%)	3 (6.52%)
	Separated	28 (2.3%)	19 (67.85%)	3 (10.71%)	6 (21.42%)
Educational level	No formal education	24 (1.1%)	22 (91.66%)	1 (4.16%)	1 (4.16%)
	Elementary school	18 (0.8%)	13 (72%)	3 (16.6%)	2 (11.11%)
	Intermediate school	48 (2.2%)	32 (66.66%)	11 (22.91%)	5 (10.41%)
	High school	577 (26.8%)	359 (64.45%)	150 (26.92%)	68 (12.21)
	Bachelor’s degree/diploma	1353 (62.8%)	878 (64.89%)	335 (24.75%)	140 (10.34%)
	Postgraduate degree	133 (6.2%)	76 (57.14%)	36 (27.06%)	21 (15.78%)
Residence	Northern region	777 (36.09%)	557 (71.68%)	164 (21.10%)	56 (7.21%)
	Southern region	466 (21.64%)	278 (62.33%)	118 (26.46%)	70 (15.70%)
	West region	414 (19.23%)	232 (56.04%)	126 (30.43%)	56 (13.53%)
	Middle region	340 (15.79%)	233 (68.53%)	75 (22.06%)	32 (9.41%)
	East region	156 (7.25%)	80 (51.28%)	53 (33.97%)	23 (14.74%)
Occupation	Education sector	414 (19.2%)	267 (64.49%)	100 (24.15%)	47 (11.35%)
	Health sector	286 (13.3%)	159 (55.59%)	90 (31.46%)	37 (12.94%)
	Private companies	251 (11.7%)	180 (71.71%)	50 (19.92%)	21 (8.37%)
	Military service	165 (7.7%)	131 (79.39%)	25 (15.15%)	9 (5.45%)
	Unemployed	918 (42.6%)	565 (61.54%)	245 (26.69%)	108 (11.76%)
	Retired	119 (5.5%)	78 (65.54%)	26 (21.85%)	15 (12.61%)
Income per month	Less than 5000 SAR (1333 USD)	1089 (50.6%)	689 (63.27%)	280 (25.71%)	120 (11.02%)
	5000-10000 SAR (1333-2666 USD)	514 (23.9%)	348 (67.70%)	120 (23.35%)	46 (8.95%)
	More than 10000 SAR (2666 USD)	550 (25.5%)	343 (62.36%)	136 (24.73%)	71 (12.91%)
Personal history of COVID-19 infection	Yes	907 (42.13%)	564 (62.18%)	229 (25.25%)	114 (12.57%)
	No	1246 (57.87%)	816 (65.49%)	307 (24.64%)	123 (9.87%)
Family history of COVID-19 infection	Yes	1453 (67.49%)	885 (60.91%)	393 (27.05%)	175 (12.04%)
	No	700 (32.51%)	495 (70.71%)	143 (20.43%)	62 (80.86%)

Without categorization, the study revealed that only 595 (27.6%) showed high awareness of all symptoms and most of them had low awareness (Table 3). Similarly, their awareness of the risk factors (415; 19.3%) showed low awareness, and 1738 (80.7%) high awareness.

**Table 3:** Overall level of awareness toward symptoms and risk factors of post-COVID-19 syndrome among participants This table represents the data in N: number with its percentage (%).

Awareness level	N (%)
High awareness level toward symptoms	595 (27.6%)
Low awareness level toward symptoms	1558 (72.4%)
High awareness level toward risk factors	415 (19.3%)
Low awareness level toward risk factors	1738 (80.7%)

The participants showed high awareness toward smell disturbance (1206 - 56.0%) while awareness toward cough was the lowest (555; 25.8%) as compared with other symptoms (Table 4). Awareness toward headache was 941 (43.7%), shortness of breath was 898 (41.7%), fatigue was 850 (39.5%), attention disorders was 701 (32.6%), muscle pain was 638 (29.6%), hair loss was 506 (23.5%) and 312 (14.5%) didn't know about these symptoms.

**Table 4:** Level of awareness toward symptoms of post-COVID-19 syndrome among participants This table represents the data in N: number with its percentage (%).

Symptoms	Aware	Unaware
Headache	941 (43.7%)	1212 (56.3%)
Fatigue	850 (39.5%)	1303 (60.5%)
Attention disorders	701 (32.6%)	1452 (67.4%)
Hair loss	506 (23.5%)	1647 (76.5%)
Shortness of breath	898 (41.7%)	1255 (58.3%)
Smell disturbance	1206(56.0%)	947 (44.0%)
Chest pain	532 (24.7%)	1621 (75.3%)
Muscle pain	638 (29.6%)	1515 (70.4%)
Cough	555 (25.8%)	1598 (74.2%)
I don’t know	312 (14.5%)	

Concerning the level of awareness toward the risk factors, the study showed that most of the participants were not aware of the risk factors of PC19S (1738; 80.7%) while only 415 (19.3%) showed awareness (Table 5). The highest awareness was seen toward old age (1326; 61.6%) followed by asthma (1048; 48.7%), smoking (963; 44.7%), diabetes mellitus (754; 35.0%), obesity (677; 31.4%), COVID-19 infection (619; 28.8%), hypertension (518; 24.1%), and the lowest awareness was toward female sex (194; 9.0%).

**Table 5:** Level of awareness toward risk factors of post-COVID-19 syndrome among participants This table represents the data in N: number with its percentage (%).

Risk Factor	Aware	Unaware
Old age	1326 (61.6%)	827 (38.4%)
Smoking	963 (44.7%)	1190 (55.3%)
Diabetes Meletus	754 (35.0%)	1399 (65.0%)
Obesity	677 (31.4%)	1476 (68.6%)
Female sex	194 (9.0%)	1959 (91.0%)
Hypertension	518 (24.1%)	1635 (75.9%)
Asthma	1048 (48.7%)	1105 (51.3%)
Hospitalization due to COVID-19 infection	619 (28.8%)	1534 (71.2%)
I don’t know	256 (11.9)	

The sources of information (Table 6) about PC19S according to the participant's responses were as follows: Saudi Ministry of Health-related resources was the source for more than two-thirds of them (1487; 69.07%), media for about two-thirds of them (1388; 64.47%), relatives (708; 32.88%), and others only 23 (1.07%).

**Table 6:** Source of information This table represents the data in N: number with its percentage (%).

Source	Yes	No
Media	1388 (64.47%)	765 (35.53%)
Ministry of Health-related resources	1487 (69.07%)	666 (30.93%)
Relatives	708 (32.88%)	1445 (67.12%)
Other	23 (1.07%)	2130 (98.93%)

## Discussion

The COVID-19 pandemic continues to pose a global threat [23,24], affecting populations across all continents. Incidence rates vary, estimated to range from 10% to 35% but can be as high as 85% among hospitalized patients [25]. In Saudi Arabia, the prevalence of COVID-19 symptoms (PC19S) also shows significant variation across different regions, reaching levels as high as 96% [11]. Given these statistics, there is a pressing need to assess public awareness of COVID-19 symptoms and risk factors among the Saudi population to effectively plan and execute targeted awareness campaigns. This study explores the level of awareness among Saudis regarding symptoms commonly associated with COVID-19. It’s important to note that the terminology and understanding of COVID-19 symptoms are continuously evolving [26], and thus, the study focuses on commonly recognized symptoms while acknowledging that additional symptoms may exist.

As recorded in this study the distribution of the demographic data of the participants showed a higher percentage of those aged 18-36 (66.37%), females (51.93%), single (48.4%), bachelor/diploma holders (62.8%), unemployed (42.6%), and income less than 5000 SAR (1333 USD)/month (50.6%), as these groups are more active, more linked with social media, and more concerned with such issues. Those living in the northern region showed a higher percentage (36.09%) than those living in other regions. Participants who have one of their families infected with COVID-19 have a higher percentage (67.49%) than others, as they had experienced the COVID-19 infection.

The study showed low awareness of participants toward both symptoms and risk factors (72.4% and 80.7%, respectively), which is consistent with the findings reported by Bogale KA in a study conducted in Ethiopia [27]. Another study conducted among females in Egypt revealed that 80% of the participants had unsatisfactory levels of knowledge of PC19S, including risk factors and symptoms [28]. This low awareness might be because PC19S is a newly emerging disease entity in addition to the absence of planned awareness development programs. Awareness of smell disturbance was the highest at 56.0%, as it is the most common COVID-19 symptom among the Saudi population.

Limitations

The cross-sectional study design might have constrained the associations between the explored factors. Sociocultural and religious barriers influenced data collection, impacting the accuracy of the data and equal participation opportunities. Consequently, we opted for a self-administered online questionnaire instead of an interviewer-administered one to reach a broader segment of the community, including illiterate individuals and those from lower socioeconomic backgrounds. However, this approach introduced potential biases that limit the generalizability of the results. Additionally, distributing the questionnaire through social media platforms like WhatsApp may have missed individuals with lower educational levels and socioeconomic statuses.

## Conclusions

The COVID-19 pandemic had a significant impact on all aspects of human life, and curing acute infection is not the end of the disease, as it may cause what is known today as post-COVID-19 syndrome. Therefore, raising awareness of PC19S risk factors and symptoms is important for managing this condition. The results of this study revealed that the majority of the participants demonstrated low awareness of the symptoms and risk factors of PC19S, which needs a continuous effort to raise the population's awareness of this health-threatening condition. Awareness programs need to be planned, organized, and conducted. Specific population groups need to be targeted for a well-planned and structured educational program to improve the level of awareness. Future studies are needed to follow the status of PC19S and population awareness of its risk factors and symptoms.

## Appendices

Consent

We are medical students from Bisha University and we are going to do research about " Awareness of post-COVID-19 syndrome among the general population of the kingdom of Saudi Arabia " and we need your cooperation to realize our objectives. we would like to request your participation by answering the questionnaire.

This information will not share with anyone at all and the respondent will not be identified and the respondent has the right to withdraw at any moment.

If you agree to participate please choice yes.

Yes      ⃝

No      ⃝

 If you need anything you can contact me through samealalbe2@gmail.com

Thank you

Questionnaire form used during the study.​

1- Age? 

18-36      ⃝

37-56     ⃝

+ 56     ⃝

2-Gender?

   ⃝ Male               ⃝ Female 

3- Nationality?

   ⃝Saudi.         ⃝ Non Saudi

4-Marital status?

   ⃝Single.                      ⃝Married.                   ⃝Divorced.                      ⃝Widowed               ⃝ Separated

5-Educational level?

   ⃝Non formal education     ⃝Elementary school     ⃝Intermediate school     ⃝High school. 

   ⃝ Bachelor's degree / diploma.            ⃝ Postgraduate degree    

6-District:*

    ⃝Middle area      ⃝West area     ⃝ East area      ⃝Northern area      ⃝Southern area

7-Where do you work?

   ⃝ Military service      ⃝ Health worker      ⃝ Education service    ⃝Private sector      ⃝  Retired      ⃝ not employed   

8- Your income per month? If there.

   ⃝ Less than 5000 SAR  (1333 USD)    ⃝ 5000-10000SAR (1333-2666 USD)      ⃝ More than 10000  SAR (2666 USD)

Measuring awareness of post-Covid 19 syndrome

9- Have you ever heard about post covid syndrome?

    ⃝Yes.       ⃝No.      ⃝ Maybe

10-What resource of information about COVID19 have you ever used? multiple answer

   ⃝Media.           ⃝Ministry of health-related resources.       ⃝ Relatives     Others     

11-Do you think one of the following is one of post COVID-19 syndrome symptoms: (choose      ⃝Yes.       ⃝No       ⃝ I don’t know)

1- Headache:                                                    ⃝Yes.               ⃝No                          

2- fatigue:                                                         ⃝Yes.                ⃝No                             

3- attention disorders:                                 ⃝Yes.                ⃝No                              

4-Hair loss:                                                      ⃝Yes                 ⃝No                           

5-Shortness of breath:                                                      ⃝Yes                 ⃝No                        

6- Smell disturbance :                           ⃝Yes                  ⃝No                              

7-Chest Pain:                                                  ⃝Yes                 ⃝No                           

8- Muscle pain :                           ⃝Yes                 ⃝No   

9-Cough:                                                      ⃝Yes                  ⃝No               

10-I don’t know

12- Which of  the following is a risk factor for Post covid syndrome? You can choose more than one answers

1- Old age:                                                        ⃝Yes.                  ⃝No                           

2- Smoking:                                                      ⃝Yes.                 ⃝No                

3- Diabetes:                                                       ⃝Yes.                 ⃝No                            

4-Obesity:                                                         ⃝Yes.                 ⃝No               

5-Female gender:                                                     ⃝Yes                 ⃝No                        

6-Hypertension:                                                ⃝Yes                 ⃝No                    

7-Asthma:                                                         ⃝Yes                  ⃝No                     

8-Hospitalization due to corona infection:       ⃝Yes                  ⃝No

9- I don’t know                                                 ⃝       

13- Have you been infected with the covid 19? 

   ⃝Yes.       ⃝No.

14- have any of your family members been infected with the COVID 19? ⃝Yes.       ⃝No.
